# Milestones of Hematopoietic Stem Cell Transplantation – From First Human Studies to Current Developments

**DOI:** 10.3389/fimmu.2016.00470

**Published:** 2016-11-09

**Authors:** Mateja Kralj Juric, Sakhila Ghimire, Justyna Ogonek, Eva M. Weissinger, Ernst Holler, Jon J. van Rood, Machteld Oudshoorn, Anne Dickinson, Hildegard T. Greinix

**Affiliations:** ^1^BMT, Department of Internal Medicine I, Medical University of Vienna, Vienna, Austria; ^2^Department of Internal Medicine III, University Hospital of Regensburg, Regensburg, Germany; ^3^Transplantation Biology, Department of Hematology, Hemostasis, Oncology and Stem Cell Transplantation, Hannover Medical School, Hannover, Germany; ^4^Department of Immunohematology and Blood Transfusion, Leiden University Medical Center, Leiden, Netherlands; ^5^Hematological Sciences, Institute of Cellular Medicine, Newcastle University, Newcastle upon Tyne, UK; ^6^Division of Hematology, Medical University of Graz, Graz, Austria

**Keywords:** hematopoietic stem cell transplantation, milestones, conditioning, HLA typing, stem cell source

## Abstract

Since the early beginnings, in the 1950s, hematopoietic stem cell transplantation (HSCT) has become an established curative treatment for an increasing number of patients with life-threatening hematological, oncological, hereditary, and immunological diseases. This has become possible due to worldwide efforts of preclinical and clinical research focusing on issues of transplant immunology, reduction of transplant-associated morbidity, and mortality and efficient malignant disease eradication. The latter has been accomplished by potent graft-versus-leukemia (GvL) effector cells contained in the stem cell graft. Exciting insights into the genetics of the human leukocyte antigen (HLA) system allowed improved donor selection, including HLA-identical related and unrelated donors. Besides bone marrow, other stem cell sources like granulocyte-colony stimulating-mobilized peripheral blood stem cells and cord blood stem cells have been established in clinical routine. Use of reduced-intensity or non-myeloablative conditioning regimens has been associated with a marked reduction of non-hematological toxicities and eventually, non-relapse mortality allowing older patients and individuals with comorbidities to undergo allogeneic HSCT and to benefit from GvL or antitumor effects. Whereas in the early years, malignant disease eradication by high-dose chemotherapy or radiotherapy was the ultimate goal; nowadays, allogeneic HSCT has been recognized as cellular immunotherapy relying prominently on immune mechanisms and to a lesser extent on non-specific direct cellular toxicity. This chapter will summarize the key milestones of HSCT and introduce current developments.

## Introduction

Seven decades ago, scientists working on the Manhattan Project in the United States discovered that the hematopoietic system was the most radiation-sensitive tissue. In 1945, the plutonium and the atom bomb ended World War II by striking Japan with over 200,000 fatalities. Subsequently, scientists began to explore ways of protecting humans from irradiation. In 1949, Jacobson and colleagues made the observation that mice were able to survive otherwise lethal irradiation when their spleen was exteriorized and protected from irradiation (1). Furthermore, intraperitoneal injection of spleen cells (1) or infusion of bone marrow (BM) cells (2) achieved the same protective effect resulting in animals’ survival. In the late 1950s, engraftment of donor-derived BM cells in lethally irradiated mice and dogs was reported (3, 4). Later on, with the concept of using irradiation for therapeutic elimination of leukemia, the use of conditioning regimens for successful transplantation was introduced into clinic. Thomas performed the first ever BM transplantation (BMT) for acute leukemia patients. He conditioned the patients with total body irradiation (TBI) and high-dose chemotherapy to get rid of the underlying disease and then infused BM, which led to hematological reconstitution (5). Unfortunately, major complications including graft failure, graft rejection, graft-versus-host disease (GvHD), and/or death from opportunistic infections led to poor transplant outcomes, and no patients who were transplanted in the late 1950s and early 1960s survived.

In 1958, van Rood and colleagues recognized that, during pregnancy, about one-third of women formed antibodies against human leukocyte antigens (HLA), which made it possible to unravel the genetics of HLA (6, 7). Thereafter, numerous studies elucidated the role of these antigens in hematopoietic stem cell transplantation (HSCT) leading to an improved understanding of the importance of HLA typing and thus, improved donor selection strategies. In 1968, van Bekkum, Balner, and colleagues (8) had successfully developed a HSCT protocol in monkeys and shared that information not only in the Netherlands but also with Good and coworkers in the United States. That same year, three patients, two in the United States and one in the Netherlands, all suffering from a congenital immune deficiency, were succesfully transplanted with hemopoietic stem cells from a HLA-identical sibling donor (9). In 1972, Thomas and colleagues reported the first experience with allografting for severe aplastic anemia (SAA) (10). In the following years, more centers were able to perform allogeneic HSCT successfully in patients with hematologic malignancies including acute leukemia.

In the 1970s, a major concern was the limitation of allogeneic grafting to HLA-identical sibling pairs. Only about one-fourth of the patients in need had a suitable stem cell donor. In 1979, Hansen and colleagues performed the first successful marrow graft from an unrelated donor (URD) for a patient with leukemia (11). After establishing URD registries in numerous countries and their cooperation under the umbrella of the BM donors worldwide (BMDW), an increasing number of patients have received allogeneic HSCT.

The use of peripheral blood stem cells (PBSC) or cord blood (CB) instead of BM for HSCT has meantime become a routine part of transplantation. Until the early 1919s, only myeloablative (MA) conditioning, including cyclophosphamide (CY), busulfan (BU), and/or TBI, was in clinical use (12, 13). In the mid-1990s, introduction of fludarabine (FLU) (14, 15) and reduction of doses of alkylating agents (16) as well as TBI dose (17), established non-MA (NMA) or reduced-intensity conditioning (RIC).

In the following sections, we will describe the current developments in allogeneic HSCT focusing on conditioning therapies, donor selection, and stem cell sources.

## Conditioning Therapy for HSCT

For successful HSCT, it is necessary that the incoming donor stem cells have sufficient graft space and support for proliferation and differentiation. Therefore, the existing host stem cells must be eradicated from the host stem cell niche in the BM, or suppressed from growth in order for donor stem cells to engraft adequately. It is also crucial that recipients are immunocompromised to prevent rejection of the incoming donor cells by the host immune system. The pretransplant conditioning regimen suppresses and functionally eradicates the host immune system and thus allows donor stem cells to home in the BM microenvironment without the risk of graft rejection. Finally and most importantly, the conditioning therapy eradicates the underlying malignant disease. This provides long-term disease control by reducing leukemic cells to a minimum, which allows final elimination by graft-versus-leukemia (GvL) effects. An exception to this rule due to a deficiency in their own immune system are infants suffering from severe combined immunodeficiency (SCID) (18) and patients with SAA with an identical twin donor who may be grafted without conditioning therapy (19).

### Types of Conditioning Regimens

Many different conditioning treatments exist, but a generally accepted definition is of two types: MA conditioning and NMA/reduced-intensity conditioning (19).

Myeloablative conditioning is of high-dose intensity consisting of a single agent or combination of agents that eradicate the patient’s hematopoietic cells in the BM and induce long-lasting trilineage aplasia. This strategy includes TBI and/or alkylating agents at doses that will not allow autologous hematologic recovery resulting in profound pancytopenia within days from the time of administration (19). Pancytopenia is life-threatening and fatal unless patients’ hematopoiesis is restored by infusion of hematopoietic stem cells (HSCs). TBI has been the primary therapeutic modality for allogeneic HSCT for patients with hematological malignancies. TBI has retained wide usage during the last decades due to its excellent immunosuppressive properties, activity against a wide variety of malignancies including ones refractory to chemotherapy, penetration of sanctuary sites such as the central nervous system (CNS) and the relative lack of non-hematologic toxicities when given at high doses. Most frequently, fractionated TBI of 12–14 Gy given over 3–4 days has been combined with CY at a dose of 120 mg/kg body weight (BW) administered over 2 days (20) as initially used for successful BMT in the late 1970s (13). Since patients with lymphoma previously given dose-limiting local radiotherapy to the mediastinum experienced a high incidence of fatal interstitial pneumonitis syndrome (IPS) following TBI (21), non-TBI-containing conditioning regimens were explored. Chemotherapy regimens also allowed to avoid the long-term sequelae of TBI including cataracts, sterility, growth, and developmental problems in children and secondary malignancies such as myelodysplasia (MDS) (22). BU is an alkylating agent with profound MA properties and marked activity against a variety of malignancies. A regimen of BU at a dose of 4 mg/kg/day for 4 days combined with CY at a dose of 120 mg/kg BW has been widely administered for the treatment of malignant and non-malignant diseases followed by allogeneic HSCT (12, 20). Few studies compared chemotherapy regimens with TBI-based conditioning. Two randomized studies demonstrated the equivalency of BU/CY and CY/TBI in patients with chronic myeloid leukemia (CML) in chronic phase receiving HLA-identical allografts (23, 24). One randomized study in patients with acute myeloid leukemia (AML) given HLA-identical transplants showed superiority for CY/TBI conditioning due to a lower relapse rate (25).

Although MA conditioning therapy provides rapid hematopoietic engraftment of donor cells, it also causes myelotoxicity, considerable morbidity, and mortality (20). Tissues containing proliferating cells such as gonads, hair follicles, oral mucosa, and the gastrointestinal (GI) tract are most susceptible, followed by the lung and other organs such as liver and, to a lesser extent, the renal and cardiac system. Besides mucositis, nausea, diarrhea, peripheral neuropathies, alopecia, and skin rash have been reported after MA conditioning. High-dose BU has been associated with interstitial pneumonitis, hepatic sinusoidal obstructive syndrome (26), and increased risk of chronic GvHD (27). The endothelial system has been increasingly recognized as an additional highly sensitive target, and this may explain some of the observed other organ toxicities (28).

Non-myeloablative conditioning can be defined as a regimen that will cause minimal cytopenia, little early toxicity, and does not require hematopoietic stem cell support (17, 19). Nevertheless, NMA conditioning regimens are immunosuppressive to the extent that, when followed by granulocyte-colony stimulating factor (G-CSF) mobilized PBSC or BM infusion, donor lymphohematopoietic cells can engraft with at least mixed donor/recipient chimerism (29). The final elimination of host hematopoiesis is then achieved by graft-versus-hematopoietic and GvL effects of the donor immune cells resulting eventually in full donor chimerism (17). Since Storb and colleagues demonstrated in the dog model that 2 Gy of TBI in combination with systemic immunosuppression allowed establishment of stable mixed hematopoietic chimerism after BM infusion of a DLA-identical littermate (30), low dose TBI at a dose of 2 Gy on the day of graft infusion has become a well-established NMA regimen (17, 20). Furthermore, low dose TBI has been combined with FLU at a dose of 90 mg/m^2^ over 3 days (17, 20). The Stanford group combined total lymphoid irradiation of 8–12 Gy delivered over 11 days and antithymocyte globuline (ATG) administered over 5 days in order to facilitate the presence of natural killer/T cells that suppress GvHD, but retain GvL effects (31).

Non-myeloablative conditioning regimens usually exert minor antitumor effects and rely mainly on the subsequent GvL effects of the reconstituted donor immune cells for eradication of the underlying disease.

Reduced-intensity conditioning regimens try to fill the gap between MA and NMA conditioning therapies. The concept of RIC is based on the idea of preventing the high toxicity and mortality associated with MA conditioning regimens in patients with advanced age or relevant comorbidities but providing sufficient immunoablation to prevent graft rejection (20). The goal of RIC is not always complete tumor eradication and thus complete destruction of host hematopoiesis but sufficient control of the underlying disease by cytotoxic therapy followed by the immune-mediated effects of donor graft cells (20). Although intensity of regimens applied vary considerably, all investigators aimed at replacing cytotoxic components of the conditioning regimen with less toxic, but immunosuppressive, agents to enable hematopoietic engraftment. A commonly used RIC regimen consists of FLU at a dose of 125–150 mg/m^2^ administered over 5 days in combination with melphalan at a dose of 100–140 mg/m^2^ given over 2 days showing efficacy in patients with AML and MDS (32). Slavin and colleagues reported a regimen consisting of FLU, BU, and ATG in patients both with hematologic malignancies as well as genetic disorders resulting in neutropenia and complete or partial donor chimerism in all patients (16). A sequential regimen of cytoreduction with FLU, cytarabine, and amsacrine followed by 3 days of rest and then 4 Gy of TBI, ATG, and CY (FLAMSA regimen) achieved promising results in patients with high-risk AML and MDS including ones with primary refractory disease and adverse risk cytogenetics (33). Subsequent replacement of TBI with BU further improved outcomes (34).

During the last few years, a variety of new agents have been introduced for RIC therapies including other alkylating agents such as high-dose treosulfan, clofarabine, or thiotepa in order to improve patients’ outcome by reducing relapse rates in individuals with advanced disease stages prior to HSCT (35).

Table 1 summarizes the currently and most frequently used conditioning regimens for allogeneic HSCT.

**Table 1:** **Frequently used conditioning regimens in various transplant centers worldwide**.

Intensity	Regimen	Comments
Myeloablative	CY/TBI	Profound pancytopenia, require stem cell support, substantial non-hematological toxicities
	BU/CY	
Non-myeloablative	FLU/TBI	Minimal cytopenia, do not require stem cell support
	TLI/ATG	
	Low dose TBI	
Reduced intensity	FLU/MEL	Intermittent cytopenia, reduced non-hematological toxicities
	FLU/BU	
	FLU/CY	

*CY, cyclophosphamide; TBI, total body irradiation; BU, busulfan; FLU, fludarabine; TLI, total lymphoid irradiation; ATG, antithymocyte globulin; MEL, melphalan*.

### Selection of Conditioning Therapy in HSCT

There is, as yet, no standard decision-making criteria for choosing a conditioning regimen for HSCT. Due to the scarcity of available direct comparative data from randomized clinical trials, assessing the efficacy of the various conditioning treatments is difficult. Before making a choice for a given patient, clinicians should consider relevant comorbidities, disease status, patient’s age, risk of rejection, and risk of relapse. In many diseases, MA conditioning therapy achieves a higher control of underlying malignancy, but this is at the risk of increased toxicity and higher incidence of transplant-related mortality (TRM). In contrast, RIC regimens have been associated with a higher relapse risk especially in patients with advanced stage of disease (36–38). Dreger and colleagues reported that RIC contributed to 18% of 1-year TRM (39), while MA conditioning generally contributes to over 30% of 1-year TRM, respectively (40).

In a multicenter retrospective study, Martino and colleagues reported the outcome of 836 patients receiving HLA-identical sibling donor transplants with either MA or RIC therapy (41). They observed that the 3-year relapse rate was significantly increased after RIC whereas 3-year NRM was decreased in RIC compared to MA conditioning with a similar rate of overall survival in both groups (41). This suggests that RIC is promising regarding early NRM but at the cost of disease relapse.

New tools for risk assessment before allogeneic HSCT such as the hematopoietic cell transplantation-specific comorbidity index have been used for valid and reliable scoring of pretransplant comorbidities that have predicted non-relapse mortality (NRM) and survival in large patient cohorts (42). These pretransplant assessments aim to improve HSCT outcomes by allowing the selection of conditioning intensity based on the patients’ comorbidity index.

Relapse has remained the major cause of mortality after HSCT. Peritransplant and posttransplant strategies to reduce the relapse risk have been discussed by various investigators and research groups (43, 44). So far, available clinical interventions are limited including timely reduction of systemic immunosuppression and prophylactic administration of donor lymphocyte infusions (DLI) (33, 44–46). In patients with high-risk AML and MDS, adjuvant DLI after RIC according to the FLAMSA protocol resulted in significantly improved 7-year survival and lower relapse rates compared to control HSCT patients not given additional DLIs (45). The German Lymphoma group investigated rituximab or no additional therapy in patients with relapsed or refractory lymphoma starting 21 days after allogeneic HSCT (47). Peggs and colleagues administered DLI for mixed chimerism after HSCT with RIC achieving full donor status in 19 of 22 patients (86%) with Hodgkin’s lymphoma (46). Of note, 4-year relapse incidence was 5% in these patients. Targeted tyrosine kinase inhibitors including sorafenib, sunitinib, and midostaurin have been used pre- and posttransplant in patients with AML as relapse treatment or maintenance therapy for prevention of relapse (48). Another strategy consists of posttransplant monitoring of CD34^+^ donor cell chimerism in patients with AML and azacytidine treatment for patients with a decline of CD34^+^ donor cells below 80% (49).

### Conditioning-Mediated Inflammation and GvHD

After administration of any conditioning therapy, but especially prominent after MA and RIC in contrast to NMA regimens, the major finding is epithelial damage caused by chemotherapeutic drugs and TBI leading to release of pro-inflammatory cytokines such as tumor necrosis factor (TNF)-α and interleukin-1 (IL-1) and resulting in the so-called “cytokine storm” (50). Endotoxins such as lipopolysaccharides (LPS) are also translocated across the damaged intestinal mucosa, resulting in a further activation of the host’s innate immune system and further cytokine release (51). A whole set of damage-associated molecular patterns (DAMPs) released from damaged cells such as uric acid and ATP and various pathogen-associated molecular patterns (PAMPs) released by the microbiota contribute to this activation (52). The signals generated cause activation of host antigen-presenting cells (APCs) such as dendritic cells (DC) (53) and increased presentation of HLA major and minor antigens. As a result, naive donor T-cells are recruited, activated, and expanded leading to the interaction with host APCs. At this stage, DCs initiate GvHD and prime naive T-cells (53). Recipient’s hematopoietic APCs activate donor CD8^+^ T-cells while, in the gut, non-hematopoietic APCs can activate donor CD4^+^ T-cells for the induction of GvHD (54). In this way, conditioning therapy can mediate tissue damage leading to donor T-cell expansion and attack on target organs (preferentially gut) leading to acute and/or chronic GvHD.

Couriel and colleagues evaluated the influence of MA and NMA regimens in 137 patients undergoing HLA-identical sibling donor transplantation (55). They observed significantly higher incidences of grades II–IV acute GvHD in patients given MA conditioning therapy. Furthermore, the cumulative incidence of chronic GvHD was 40% higher in patients receiving MA conditioning when compared to NMA. These results suggested that MA conditioning was not only myelotoxic but also accounted for profound higher incidences of both acute and chronic GvHD (55). Similar results were observed by Mielcarek and coworkers (56).

It can be noted that, currently, there is no best conditioning regimen available that can ensure disease-free survival (DFS) of patients after HSCT. Choice of conditioning therapy used prior to transplantation highly depends on recipient age, underlying disease, and disease status prior to HSCT, relevant comorbidities, and type of donor (matched or mismatched; related, or unrelated). MA conditioning is perhaps preferred for younger patients, and RIC may be given to patients whose underlying disease has been well controlled. A choice among various conditioning regimes is largely based upon center experience. However, randomized clinical trials comparing different conditioning therapy intensities are highly warranted to increase the level of evidence for choosing the appropriate pretransplant treatment wisely in order to allow long-term DFS with good quality of life. In addition, a standardized developed therapy worldwide, or even between European centers, would greatly facilitate the evaluation of biomarkers predicting outcome and response to therapy. This would further improve transplant results in the future.

## Importance of the HLA Region

### HLA-Typing Techniques

Improvements in HSCT would not have been possible without the significant progress made in the understanding of the HLA system and the development of HLA typing techniques. The major HLA antigens essential for immune responses are HLA-A, -B, -C, -DR, -DQ, and -DP, which are encoded by polymorphic genes in the human genome, with 1–1543 alleles per locus (for the most up to date number of HLA alleles reported in IMGT-HLA^1^). The remarkable allelic polymorphism makes HLA typing very challenging (57). The pioneering work of HLA typing was carried out with serological and cellular assays. Serological techniques started with agglutination, but were soon based on complement-dependent cytotoxicity, cell cultures in mixed lymphocyte reactions, and cell-mediated cytolysis. One of the most important drawbacks of those methods is the need for viable cells expressing surface antigens. Over the years, several improvements were made to the serological techniques culminating in the development of the Terasaki microlymphocytotoxicity test (58). After modifications, it is still in use today, especially to clarify the absence of some “null alleles” (variants affecting expression of protein) or to decrease the number of primers or probes in DNA-based tests (59).

In the 1980s, molecular techniques were introduced into HLA typing, namely, restriction fragment length polymorphism (RFLP). Amplified DNA was digested with restriction enzymes to generate specific restriction patterns, thus leading to the identification of alleles according to the pattern. Although RFLP allowed for typing with higher sensitivity and specificity than serological methods, the procedure was still very labor-intensive and did not replace serological typing (57). Further development of PCR technologies and Sanger sequencing provided new options in the field of HLA typing, such as sequence-specific oligonucleotide probes (SSOP), sequence-specific priming (SSP), and sequencing-based typing (SBT). The SSOP system in the most practical format (reverse SSOP) involved PCR amplification of the target sequence labeled with biotinylated primers followed by hybridization with the immobilized sequence-specific probes, incubation with streptavidin conjugated to an enzyme and chromogenic substrate (60). The idea of SSOP typing was also adopted for the flow cytometry technology Luminex by changing immobilization on nylon membrane to microbeads and colorimetric to fluorescence detection technology. This allowed faster, reliable, and automated typing (61). SSP typing was the alternative and the complementary system to SSOP typing, developed based on the extension of the 3′ ends of primers, which were either matched or mismatched with the target sequence. The results of SSOP and SSP typing are considered as “low” and “intermediate” HLA resolution typing (57). Low resolution (on antigen level) and also called “2-digit typing” corresponds to the identification of broad families of alleles that cluster into serotypes (e.g., A*02). It is thus, the equivalent of serological typing (A2) (62). High-resolution (HR) typing is on an allele level and allows identification of the set of alleles encoding the same protein sequence for the region of the antigen-binding site of the HLA molecule. Alleles that are not expressed as cell surface molecules are excluded. Intermediate level is the level of resolution in between high- and low-resolution (63). SBT, which is the combination of DNA amplification and direct sequencing, provided HR HLA typing. However, ambiguity at the allelic level (linked polymorphic sequence can be outside the typed region) or genotype ambiguity (inability to establish whether linked polymorphisms are on the same -cis or different -trans allele coming from the father or the mother) still remains an important problem. In order to deal with that issue, scientists included additional exons for typing or investigated preliminary/additional typing methods to HR typing, e.g., SSP (method, which can distinguish cis/trans ambiguities) (57). Over the last few years, the breakthrough in HLA typing was the development of the next-generation sequencing (NGS) technology, which offers HR and high-throughput typing. However, it requires complex sample preparation including elaborate library preparation and sample enrichment steps and considerable bioinformatics resources for data analysis. Recently, several labs have applied NGS to genotype highly polymorphic HLA genes using different strategies of amplification, library preparation, platforms for sequencing, and sequence analysis approaches to enhance sequencing coverage and resolve ambiguities (64–66). There are still some limitations to overcome, but it is highly probable that NGS will soon become the routine method for HLA typing. Thus, in the near future, centralized typing facilities could offer reasonably priced NGS-based typing when large numbers of samples can be processed in a more automated fashion.

All described methods of HLA typing are shown in Table 2.

**Table 2:** **Comparison of HLA Typing Techniques**.

Method	Benefits	Drawbacks
Serological	Preliminary or supportive method for molecular assays; fast and cheap	Low resolution; requires viable cells; poor reagent supply in the past, labor intense, not the current standard
Cellular	Used for HLA class II typing until approx. 2000	Low resolution; requires viable cells; labor intense, but informative; rarely used currently
RLFP	Used for HLA class II typing until approx. 2000	Low resolution; labor intense; did not replace serological methods; rarely used currently
SSOP	Involved in preliminary typing used today	Low or intermediate resolution; limited to previously known polymorphisms; restricted to selected exons
SSP	Nowadays used to distinguish cis/trans ambiguities	Low or intermediate resolution; limited to previously known polymorphisms; restricted to selected exons
SBT	High resolution	Does not distinguish cis/trans ambiguities; restricted to selected exons
NGS	High resolution; high-throughput typing; increases rate of resolved ambiguities	Complicated workflow and data analysis, novel technique, could become reasonably priced when used in centralized facilities

*HLA, human leukocyte antigen; RLFP, restriction fragment length polymorphism; SSOP, sequence-specific oligonucleotide probes; SSP, sequence-specific priming; SBT, sequencing-based typing; NGS, next-generation sequencing; approx., approximately*.

### Choice of Donors for HSCT

Over the last few years, based on the outcome of many studies, identification of 10 alleles in 5 HLA loci, namely, HLA-A, -B, -C, -DRB1, and -DQB1 using HR typing has become the gold standard of URD matching in accordance with the guidelines of the European Society for Blood and Marrow Transplantation (62). In the United States, the National Marrow Donor Program Committee has recommended allele-level typing for HLA-A, -B, -C, and -DRB1 to obtain 8/8 or 7/8 allelic identity (67) questioning the importance of HLA-DQB1 matching for outcome (68, 69). Due to the low numbers of mature T-cells in CB, higher levels of HLA-incompatibility between donor and recipient are accepted. Therefore, selection of CB units is primarily based on HLA-A, -B intermediate resolution level, and DRB1 HR level. A recent study by the Center for International Blood and Marrow Transplantation Research (CIBMTR) and Eurocord reported better outcomes in single CB transplants with improved allele-level matching for four HLA loci (-A, -B, -C, and -DRB1) suggesting that CBT with three or more allele level mismatches should be avoided, due to unacceptable levels of NRM and poorer survival (70). HR typing at 4 loci and selecting CB units matched for at least 5/8 alleles also improved TRM after double CBT (71).

In the search algorithm, genotypically identical related donors are considered to be first choice based on rapid availability and the likelihood of not only major but also minor histocompatibility antigen identity. The probability of HLA identity of a sibling is 25%. In a study utilizing birth data and statistical modeling, Besse and colleagues reported considerable variation in the likelihood in families of an HLA-identical sibling donor, ranging from 13 to 51% depending upon patient age and race/ethnicity (72). Furthermore, the present 40-year decline in birth rates is expected to lead to a 1.5-fold decrease in access to an HLA-identical sibling for today’s young adults (18–44 years) when they reach the peak age for potential HSCT (61 years) compared to their contemporary counterparts (72). HLA typing of parents and siblings not only allows the identification of a potential-related donor but also reveals the distribution of haplotypes that can provide valid information whether an extended family search may be useful. HLA typing of a family is usually performed at low resolution level unless homozygosity is expected in the family requiring HR typing (59). Typing for HLA-A, -B, and -DR (6/6 matching) at low resolution enables, in most cases, determination of the paternal and maternal haplotypes present in the patient and a potential related donor (62).

For patients lacking an HLA-identical sibling donor, searches for HLA-matched donors among extended family members (grandparents, uncles/aunts, cousins, nieces, and nephews) have proven fruitful in populations where consanguineous or related marriage is common (73, 74). Otherwise, the alternative is an HLA-identical URD or a CB donor. The probability to find a matched unrelated donor (MUD) is around 30–70%, depending on the frequency of the HLA genotype in the donor registries and the patient’s ethnicity (67). HR HLA typing is performed when searching for URDs to provide in depth information on the HLA type of the recipient and the potential URD. As a consequence of HR typing and thus, more adequate donor selection, the outcomes of patients transplanted from matched URD have become comparable to patients transplanted from matched sibling donors (75).

In case of a lack of a MRD or MUD, a mismatched donor can be considered (9/10 or 7/8 alleles matched) when patients urgently need a HSCT. This includes haploidentical family donors (5–9/10 or 4–7/8 alleles matched) and mismatched CB donors (<6 alleles matched). Almost all patients have a haplotype-mismatched related donor (MMRD) available. This provides the enormous advantage of immediate access to this donor, a fact that is most important for patients suffering from acute leukemia, who cannot afford a lengthy donor search and are at risk of dying of their malignancy prior to HSCT. It also allows collection of additional donor cells for peritransplant or posttransplant cellular immunotherapy, if needed. In addition, the immediate donor availability has financial implications since costs for additional donor typing and URD search can be reduced.

Until a few years ago, the use of a haplotype MMRD was associated with a significantly higher risk of GvHD and graft rejection unless the graft was T-cell depleted (76). Recently, the post-transplant administration of CY on days +3 and +4 after infusion of unmanipulated BM cells from a haploidentical donor has resulted in improved outcome with low incidence rates of both acute and chronic GvHD (77, 78). Posttransplant CY promotes immune tolerance by selectively depleting rapidly proliferating alloreactive host and donor T-cells while sparing non-alloreactive memory T-cells, regulatory T-cells, and hematopoietic progenitor cells and thus, preventing antitumor and antimicrobial immunity (79). Whereas initial protocols contained BM as graft source, comparable outcomes with BM or PBSC as stem cell sources for HSCT from haploidentical donors have meantime been reported (80). Haploidentical HSCT with posttransplant CY provided survival outcomes comparable to HSCT with an HLA-identical sibling or URD in patients with lymphoma, AML, and ALL (81, 82). In retrospective analyses, results of haploidentical HSCT for patients with AML in remission appear to be comparable to the best results of CB transplantation (83). Prospective clinical trials comparing haploidentical HSCT to CB transplantation and HSCT from other donor sources are currently ongoing.

Interestingly, the superior outcome of the maternal graft over the paternal graft has been described in haploidentical transplants (84, 85). Van Rood and colleagues demonstrated that recipients of non T-cell depleted maternal transplants had a lower incidence of acute and chronic GvHD than recipients of paternal transplants (84). Moreover, Stern and colleagues showed that haploidentical T-cell depleted stem cell transplants from mother to child had a lower relapse rate and improved survival compared to paternal grafts (85). The explanation of the observed effects can be the fact that, during pregnancy, the fetal immune system is exposed to the non-inherited maternal antigens (NIMA), and the mother is sensitized to the fetus inherited paternal antigens (IPA), establishing bidirectional immunity, which is achieved by regulatory T-cells between mother and fetus (86). This concept is supported by the persistence of fetal microchimerism in the mothers after pregnancy (87).

### Effect of HLA Incompatibility and Other Clinical Parameters on HSCT Outcome

The effect of HLA mismatches on the outcome of HSCT depends mostly on the number of mismatches, locus of the mismatch, and direction of the mismatch (75, 88, 89). The immune reaction caused by an HLA-mismatch differs when the mismatch is: in the GvH direction – donor homozygous at mismatched loci; in the host-versus-graft (HvG) direction – recipient homozygous at mismatched loci or is bidirectional – donor and recipient heterozygous at mismatched loci. The mismatched antigen in the GvH direction may be targeted by donor T-cells and cause GvHD, a mismatch in the HvG direction may be recognized by recipient T-cells and promote graft rejection, whereas a bidirectional mismatch may affect both outcomes (88, 90). Whereas patients with hematologic malignancies may benefit from the GvL effect associated with HLA-mismatched donors, this is different for patients with non-malignant diseases requiring allogeneic HSCT where the adverse effect of GvHD is not counterbalanced by a beneficial GvL effect. Mismatched transplants for patients with non-malignant disorders are strongly associated with an increased risk of graft failure, probably also due to the increased use of T-cell depletion prior to HSCT in order to decrease harmful GvHD in those patients. The recommendation for transplantation of patients with non-malignant disorders is to use matched donors whenever possible (91, 92).

Human leukocyte antigen disparity between donor and recipient impacts on the risk of severe GvHD, graft failure, and delayed immune reconstitution (93–96). On the other hand, HLA mismatches can be tolerated in transplant settings using *in vitro* T-cell depleted grafts and permissive HLA mismatches, which do not result in worse outcome (97–99).

During the last few years, the impact of allelic mismatches in specific HLA loci on the risk of GvHD development has been investigated. Several groups have shown an association between allelic mismatches in HLA-A, -B, -C, and -DRB1 and higher rates of acute GvHD (94, 100, 101). However, limited data have been published on the impact of HLA class I and class II disparities on the incidence and severity of chronic GVHD. Interestingly, chronic GvHD was triggered mainly by mismatches in HLA class I (94, 102). Morishima and colleagues found HLA-A and/or HLA-B allele mismatches to be a significant risk factor for the occurrence of chronic GvHD (94).

Since HLA-disparity between recipient and URD is a known risk factor for GvHD, and this complication also increases the incidence of opportunistic infections after HSCT, it is difficult to investigate the impact of HLA-disparity *per se* on immune reconstitution and infectious complications. However, Maury and colleagues identified an independent association of HLA incompatibility between recipient and URD on delayed recovery of CD4^+^ T-cells and decreased T-cell proliferative responses (103). Few studies explored the impact of HLA mismatches on the rate of infections after HSCT. It has been shown that mismatched donors or URDs are independent risk factors for death due to late infection (later than 6 months after HSCT) (104). Moreover, Ljungman and colleagues reported results from a multivariate analysis indicating that recipients of mismatched family or URD grafts were more prone to develop cytomegalovirus (CMV) disease and die due to CMV-associated complications than recipients of grafts from HLA-matched sibling donors (105). In addition, Poutsiaka and colleagues observed that HLA mismatches between donor and recipient independently increased the risk of blood stream infections (106). Reasons for delayed immune reconstitution after HLA-incompatible donor HSCT may be impaired antigen presentation by APCs or impaired thymic function, since it has been previously shown that HLA mismatches negatively influence thymic-dependent T-cell reconstitution (107). However, further research on long-term immune reconstitution in the context of HLA-mismatched HSCT, especially in the adult population, is warranted.

In addition to HLA disparity, other factors are known to influence the outcome of HSCT including patient and donor age, ethnicity, and gender. The impact of patient age has been investigated by Cornelissen and colleagues in AML patients observing an adverse effect of increasing patient age on outcome due to an age-related rise of treatment-related complications (108). On the other hand, administration of RIC regimens for HSCT in older patients with AML was well tolerated and NRM at 2 years was 15% (109).

Donor age appears to be also an important factor for selecting the best donor. The data from several studies suggest that younger donor age is associated with better outcome after HSCT (110–113). Bastida and colleagues reported that patients with AML and MDS who received a graft from a donor above the age of 50 years had a worse overall survival, higher TRM, and higher relapse rates (113).

The effect of recipients’ ethnicity has been reported as additional factor affecting outcome after HSCT. A comparison of results obtained after HSCT of Caucasians, African Americans, Hispanics, and Asians showed a decreased overall survival and higher risk of treatment failure among Hispanics (114–116). These differences in the outcome after HSCT are not well understood. They might be explained by polymorphisms in cytokine genes (117) and differences in minor histocompatibility antigens (mHAs) (118). However, the evaluation of the impact of donor ethnicity and donor-recipient ethnic identity did not support drawing donor ethnicity into consideration in the donor selection algorithm (119).

Various investigators observed a higher risk for transplant-related complications including GvHD after HSCT of male recipients with female donor grafts (120, 121). Of note, risk of relapse was significantly decreased in male recipients experiencing chronic GvHD and having an antibody response to recipient HY antigen (122).

### Graft-versus-Leukemia Effect

While both GvL and GvHD are caused by major or minor histocompatibility antigen mismatches, prevention of leukemic relapse by enhancing the GvL effect is frequently limited by GvHD. It has become a major clinical issue to improve outcomes by separating GvL from GvHD effects in the field of HSCT. The role of mHAs in matched donor transplantation has been predominantly investigated in order to overcome this challenge (123).

However, few researchers have addressed the problem in terms of major HLA antigens. Kawase and colleagues identified eight mismatch combinations (two HLA-Cw and six HLA-DPB1), which were associated with decreased risk of relapse and differed from mismatches responsible for severe acute GvHD (124) Moreover, patients given grafts with these combinations of HLA-DPB1 had significantly better overall survival compared to recipients of completely matched donor/recipient pairs (124). Shaw and colleagues reported comparable data concerning the role of HLA-DPB1 mismatch and a lower risk of relapse, but this effect was accompanied by an increased risk of acute GvHD (125). A model for identification of non-permissive HLA-DPB1 mismatches by the presence of T-cell-epitope mismatching has been proposed in order to provide a clinical strategy for lowering the risk of mortality after URD transplants (98, 126). Recently, Petersdorf and colleagues revealed the mechanism leading to the higher incidence of acute GvHD in recipients of grafts mismatched for HLA-DPB1 (69). They found that the risk of GvHD was influenced by the single nucleotide polymorphism in the HLA-DPB1 region responsible for the genetic control of HLA-DP expression levels. Thus, these data need further investigation but may be helpful in the future for selection of the best donor.

In a retrospective study of single unit CB recipients, van Rood and colleagues demonstrated that patients with AML and ALL who shared one or more HLA-A, -B, or -DRB1 antigens with their CB donor’s IPAs had a significant decrease in leukemic relapse after HSCT compared with those who did not, providing indirect evidence that maternal microchimerism in CB mediates a GvL effect in CB transplantation (127).

### Role of KIR Ligand Mismatches

Killer immunoglobulin-like receptors (KIRs) are NK receptors binding to the HLA class I molecules and thus, control the activity of NK cells. There are two types of KIRs; one inhibits the ability of NK cells to kill foreign cells and the other activates NK cells (128). Apart from the broad diversity of activating and inhibitory receptors on NK cells, differences in the expression of NK cell ligands on the cell surface of target cells determine the induction or inhibition of NK cell activity. NK cell alloreactivity in patients after HSCT is directed against leukemic cells and mediated by mismatches in the graft-versus-host (GvH) direction in HLA class I molecules, which cause the incompatibility in binding to KIRs (129). There are three known KIR ligand mismatches in the GvH direction, all of which are present in donor/missing in recipient: (1) HLA-C1, (2) HLA-C2, and (3) HLA-BW4 (130). HLA and KIR genes segregate independently on different chromosomes, thus only 25% of HLA identical siblings and less than 1% of MUD are KIR identical (130). It has been demonstrated by *in vitro* studies, murine models, and several clinical studies that KIR ligand mismatches in GvH direction are important for the success of HSCT with a haploidentical donor in patients with AML. GvH NK alloreactivity was associated with significantly improved survival, favored engraftment, eradication of AML, and reduced GvHD (131, 132). These clinical observations are based on the fact that NK cells mediate clearance of (1) residual leukemia cells resulting in lower relapse rate, (2) host T-cells improving hematopoietic engraftment, and (3) host dendritic cells reducing GvHD incidence (133).

On the other hand, conflicting results were presented on the beneficial effect of KIR ligand incompatibilities and outcome after unrelated HSCT. Giebel and colleagues reported that overall survival of patients with ALL, AML, or CML, transplanted with unmanipulated grafts of MUD with KIR ligand incompatibilities, was significantly improved (134), but other studies failed to reproduce these results (135–137). The advantage of KIR ligand mismatches on survival became more pronounced, when analysis was limited to AML patients (138, 139). The discrepancies in the results of the aforementioned studies can be explained by the heterogeneity of treatment protocols and patient cohorts. However, difficulties arise in connection with KIR ligand mismatches and outcome after HSCT. In analyses, it is difficult to show advantages of KIR-ligand mismatches when mismatches in the GvH and HvG direction exist on the same HLA molecules. Strong response from alloreactive T-cells toward the incompatible HLA molecule can override the favorable effect of KIR ligand mismatch (140).

## Stem Cell Sources

For many years, BM harvested from the posterior iliac crests under general anesthesia had been used as the source of HSC for transplantation. In the 1990s, two new HSC options, namely, G-CSF-mobilized PBSCs and CB became available for clinical use. Although there are many differences between these three HSC sources, clinical results after HSCT seem to be comparable (141–143). The choice of different stem cell sources depends on age of the donor and the recipient, clinical comorbidities, as well as disease stage, and varies depending on the preferences of different centers and donors (Table 3).

**Table 3:** **Comparison of hematopoietic stem cell sources**.

Stem cell source	Benefits		Drawbacks	
	Donor	Recipient	Donor	Recipient
BM		Lower risk of GvHD	More invasive HSC collection	
PBSC	No general anesthesia for collection; less discomfort and pain	Faster hematopoietic engraftment and immune reconstitution; enhanced GvL effect		Higher risk of GvHD
CB	Non-invasive	Lower risks of GvHD and relapse; rapid availability; increased level of HLA-disparity tolerated		Lower number of HSCs; slower immune reconstitution

*BM, bone marrow; PBSC, peripheral blood stem cells; CB, cord blood; GvHD, graft-versus-host disease; HSC, hematopoietic stem cell; GvL, graft-versus-leukemia, HLA, human leukocyte antigen*.

### PBSC – Benefits for Patients and Donors

One of the major changes in HSCT was the replacement of BM by G-CSF-mobilized PBSCs (144, 145). Over the past decade, PBSCs have become the preferable stem cell source in many transplant centers, accounting for around 75% of all HSCTs performed (142, 146, 147).

Use of PBSCs holds several advantages over BM. HSC collection from peripheral blood (PB) is preferred by donors as it spares them general anesthesia and cells can be harvested in the outpatient setting (145, 148). Karlsson and colleagues analyzed 171 donors and reported significantly more prolonged pain and severe fatigue in BM donors compared to PBSC donors (149). So far, complications of growth factor administration and leukapheresis such as malignancy and stroke are no higher than those of BM collection (141). A large study in more than 9000 PBSC and BM donors demonstrated a lower risk of serious adverse events (SAE) in donors of PBSC (150). Furthermore, PBSC donors treated with G-CSF have shown no increased risk of cancer, autoimmune disease, or stroke compared with BM donors and even a lower incidence of cancer compared with the general population (150).

Besides benefits for the donors, there are advantages for the recipients as well. Faster hematopoietic engraftment and immune reconstitution have been observed in patients receiving PBSC compared to those given BM grafts (145, 151). In a clinical study using MA conditioning therapy with HLA-identical related donors, 5 and 6 days of earlier neutrophil and platelet engraftment, respectively, were observed after PBSC compared to BM grafts (152). Furthermore, after HSCT with MUD, a shorter time to absolute neutrophil count equal to 0.5 × 10^9^/L and a shorter time of platelet engraftment were reported in the PBSC compared to the BM group (153). Several other studies and a meta-analysis including eight different trials in MUD confirm these findings and showed a higher rate of engraftment in recipients of PBSC (146, 154, 155).

Moreover, high numbers of lymphocytes in the PBSC, namely, immunocompetent T-cells may enhance the GvL effect (145, 155). Unmodified PBSC grafts may contain one log more T-lymphocytes than unmodified BM grafts (156). However, these high T-cell numbers could in parallel lead to a higher risk of GvHD (155, 156), which as a consequence may have a higher mortality. An increased incidence of chronic GvHD, but no difference in acute GvHD, was observed between PBSC and BM graft recipients in HLA-matched related settings by Campregher and colleagues (157). Eapen and colleagues reported a significantly higher incidence of chronic GvHD after MA conditioning and URD PBSC infusion (158). Although mortality risks were higher in patients with chronic GvHD, both in PBSC and BM settings, and PBSC recipients had more severe chronic GvHD, there was no difference in mortality between these two graft types (158). A multicentre, randomized trial published by Anasetti and colleagues similarly reported no difference in acute GvHD and a higher incidence of chronic GvHD in PBSC recipients, but no difference in the 2-year-survival rate compared to BM recipients (142).

Progression-free survival seems to be comparable between PBSC and BM recipients, and the risk of relapse appears to be lower in patients given PBSC (142, 151, 155).

### Cord Blood – A Life-Saving Alternative

Despite the advances made in HSCT over the last decades, donor availability has remained a major obstacle and introduction of CB provided an alternative for these patients (159, 160). The first CB transplantation was performed successfully in Paris in 1988 in a pediatric patient suffering from Fanconi anemia (FA) (161). Results of CBT in adults were less favorable (160, 162). In a study by Laughlin and colleagues on 68 patients who underwent CBT, 17 died most likely as a result of the preparative regimen and 22 patients died due to an infection after HSCT (162). High death rates were attributed in part to the selection of high-risk patients, but slow myeloid engraftment could have also contributed (162). In the following years, better CB and patient selection substantially improved CBT outcome (154, 163–165). Since then, according to the BMDW database, more than 30,000 CBT have been performed and CB banks have been established around the world storing more than 600,000 CB units (160).

One of the main advantages of CB is the fact that an increased level of HLA disparity can be tolerated (166). The current standard for CB selection is donor–recipient matching at six HLA loci, namely, HLA-A, HLA-B antigen, and HLA-DRB1 allele in comparison to 8–10 loci for BM or PBSC donation (167, 168). Despite increased tolerance, HLA matching still remains, together with the cell dose infused, one of the main factors associated with improved engraftment and better survival (169, 170). The negative impact of HLA-disparity on patient outcome could be partially overcome by higher CD34^+^ cell doses for each level of HLA disparity. Better survival was demonstrated in recipients of CB grafts with two HLA-mismatches given more than 1.7 × 10^5^ CD34^+^ cells per kilogram BW than those receiving a lower dose (171). Data suggest that the CD34^+^ cell content should be the most important criterion when choosing CB grafts, followed by the degree of HLA-disparity (154). The number of total nucleated cells collected or infused should not be less than 2.5 × 10^7^/kg BW (168, 170). Indeed, the main limitation of CB is the low number of HSCs in contrast to the numbers typically present in BM or PBSC allografts (172). Since several studies reported better engraftment in recipients of higher doses of CD34^+^ cells (162, 169, 170), use of double CB units was introduced some years ago and has been proven safe, showing comparable overall outcomes as matched-related and unrelated HSCT (159, 172). Wagner and colleagues compared HSCT with one CB unit with double CB units used in children and adolescents with hematologic malignancies and observed no differences in survival, neutrophil recovery, and immune reconstitution between the two groups (173). However, recipients of single CB units achieved better platelet recovery and had a lower incidence of more severe acute GvHD and chronic GvHD (173).

Several studies have been performed in order to compare outcome of CB versus BM or PBSC transplantation. In 2004, Laughlin and colleagues compared mismatched CBT and mismatched BMT in adult patients and observed no significant differences in TRM, treatment failure, and overall mortality between these patient cohorts (174). Rocha and colleagues reported no significant differences between mismatched CBT and matched BMT regarding TRM, relapse rate, and leukemia-free survival (175). Takahashi and colleagues observed lower TRM and better DFS after CBT compared to BMT despite a higher HLA-mismatching rate in CBT recipients (176). The same group later reported no differences in TRM, DFS, and relapse rate after CBT when compared with BM and PBSC grafting (136). More recent studies support these results (143, 177), respectively. Terakura and colleagues analyzing HSCT outcomes in patients with ALL and AML reported similar OS and NRM comparing 8/8 allele-matched unrelated BMT with CBT leading to the conclusion that CB could be a preferable alternative (178).

Regarding the incidence of GvHD, Laughlin and colleagues reported higher rates of acute GvHD after MMUD marrow grafts and higher chronic GvHD rates after CBT (174), while Rocha and colleagues observed a lower risk of grades II–IV acute GvHD after CBT and a comparable incidence of chronic GvHD between CB and unrelated BM recipients, respectively (175). Others observed similar rates of severe acute GvHD after CBT and 8/8 matched BMT but higher rates after 7/8 matched BM grafting while the incidence of extensive chronic GvHD was significantly lower after CBT compared with 8/8 and 7/8 BM grafting (178).

In conclusion, CB as an alternative HSC source is comparable to BM and PBSC, and offers several advantages, namely, easier availability, higher tolerable HLA-disparity, lower risks of GvHD, and relapse. Nevertheless, limited cell numbers and slow immune reconstitution contributing to infections and impacting survival remain an obstacle. Novel strategies for improvement of hematopoietic and immune reconstitution after CBT include *ex vivo* expansion of CB cells using different cytokine combinations, intra-bone injection of cells, modification of homing, and the coadministration of mesenchymal stromal cells (159, 172).

## Conclusion and Outlook

Allogeneic HSCT has become an established curative treatment of a steadily increasing number of life-threatening hematological, oncological, hereditary, and immunological diseases. During the last decades, combined research efforts including preclinical models and clinical studies on a worldwide scale has resulted in an impressive progress in various areas of HSCT. Improved patient selection, development of improved tissue typing methods, availability of URD and CB units as HSC source, and introduction of RIC and NMA conditioning regimens has resulted in improved patients’ survival over the years. However, overall survival rates have remained at 40–50% for over two decades. Further, interdisciplinary research and team efforts are necessary to improve malignant disease eradication and further inspire survival in the future. In addition, a worldwide collective effort is necessary to standardize conditioning protocols, which would aid in improving outcomes.

Currently, cellular-based immunotherapies, which were pioneered by the development of allogeneic HSCT are gaining increasing clinical relevance for treatment of patients with hematologic malignancies. For decades, the contribution of donor’s immune cells to elimination of host tumor cells in leukemia, lymphoma, and myeloma after HSCT has been appreciated (179–181). To reduce or avoid the occurrence of GvHD that is associated with significant morbidity and mortality, more precise and effective cell-based therapies have been developed. Immune cell engineering including adoptive transfer of T-cells genetically modified to express chimeric antigen receptors (CARs) specific for a selected tumor antigen such as CD19 in B-cell malignancies have demonstrated impressive antileukemic activity in patients with ALL, lymphoma, and chronic lymphocytic leukemia (182–185). Optimizing T-cell receptor gene therapy for hematologic malignancies aims at improving the efficacy of T-cell therapies by maintaining their effector function and promoting memory. Recent gene-editing tools such as transcription activator-like effector nucleases (TALEN) and clustered regularly interspaced short palindromic repeats (CRISPR) allow deletion of endogenous T cell receptor and HLA genes leading to removal of alloreactivity and decreased immunogenicity of third-party T-cells. Talen-engineered CAR19 T-cells from a third-party donor have recently been administered to a 11-month-old girl with relapsed B-ALL after allogeneic HSCT resulting in complete cytogenetic and molecular remission (186). This represents an important scientific development toward generic off-the-shelf T-cell receptor engineered products for treatment of a larger number of patients with hematologic malignancies.

## Author Contributions

EW, EH, AD, and HG designed the review and revised it critically for important intellectual content. MJ, SG, and JO provided the draft, summarized available data, and selected the references. JR and MO reviewed the manuscript and provided important suggestions. All the authors approved the final version of the manuscript.

## Conflict of Interest Statement

The authors declare that the research was conducted in the absence of any commercial or financial relationships that could be construed as a potential conflict of interest. The reviewer RC and handling Editor declared their shared affiliation, and the handling Editor states that the process nevertheless met the standards of a fair and objective review.

## Footnotes

^1^Immunopolymorphism database (IPD) – International ImMunoGeneTics project (IMGT) database. Available from: http://www.ebi.ac.uk/ipd/imgt/hla/stats.html.
